# Predicting the Progress of Tuberculosis by Inflammatory Response-Related Genes Based on Multiple Machine Learning Comprehensive Analysis

**DOI:** 10.1155/2023/7829286

**Published:** 2023-05-16

**Authors:** Shuai Ma, Peifei Peng, Zhihao Duan, Yifeng Fan, Xinzhi Li

**Affiliations:** ^1^Hubei Key Laboratory of Tumor Microenvironment and Immunotherapy, China Three Gorges University, Yichang 443000, China; ^2^College of Basic Medical Science, China Three Gorges University, Yichang 443000, China; ^3^Department of Geriatrics, Liyuan Hospital, Tongji Medical College, Huazhong University of Science and Technology, Wuhan, Hubei 430074, China

## Abstract

**Background:**

Tuberculosis (TB), caused by the bacterium *Mycobacterium tuberculosis*, affects approximately one-quarter of the global population and is considered one of the most lethal infectious diseases worldwide. The prevention of latent tuberculosis infection (LTBI) from progressing into active tuberculosis (ATB) is crucial for controlling and eradicating TB. Unfortunately, currently available biomarkers have limited effectiveness in identifying subpopulations that are at risk of developing ATB. Hence, it is imperative to develop advanced molecular tools for TB risk stratification.

**Methods:**

The TB datasets were downloaded from the GEO database. Three machine learning models, namely LASSO, RF, and SVM-RFE, were used to identify the key characteristic genes related to inflammation during the progression of LTBI to ATB. The expression and diagnostic accuracy of these characteristic genes were subsequently verified. These genes were then used to develop diagnostic nomograms. In addition, single-cell expression clustering analysis, immune cell expression clustering analysis, GSVA analysis, immune cell correlation, and immune checkpoint correlation of characteristic genes were conducted. Furthermore, the upstream shared miRNA was predicted, and a miRNA–genes network was constructed. Candidate drugs were also analyzed and predicted.

**Results:**

In comparison to LTBI, a total of 96 upregulated and 26 downregulated genes related to the inflammatory response were identified in ATB. These characteristic genes have demonstrated excellent diagnostic performance and significant correlation with many immune cells and immune sites. The results of the miRNA–genes network analysis suggested a potential role of hsa-miR-3163 in the molecular mechanism of LTBI progressing into ATB. Moreover, retinoic acid may offer a potential avenue for the prevention of LTBI progression to ATB and for the treatment of ATB.

**Conclusion:**

Our research has identified key inflammatory response-related genes that are characteristic of LTBI progression to ATB and hsa-miR-3163 as a significant node in the molecular mechanism of this progression. Our analyses have demonstrated the excellent diagnostic performance of these characteristic genes and their significant correlation with many immune cells and immune checkpoints. The CD274 immune checkpoint presents a promising target for the prevention and treatment of ATB. Furthermore, our findings suggest that retinoic acid may have a role in preventing LTBI from progressing to ATB and in treating ATB. This study provides a new perspective for differential diagnosis of LTBI and ATB and may uncover potential inflammatory immune mechanisms, biomarkers, therapeutic targets, and effective drugs in the progression of LTBI into ATB.

## 1. Introduction

Tuberculosis (TB), caused by *Mycobacterium tuberculosis*, is a highly prevalent disease that affects approximately one-quarter of the global population, resulting in millions of deaths each year [1, 2]. While a majority of these cases are latent tuberculosis infections (LTBI), 5%–10% of infected individuals may develop active tuberculosis (ATB) [3], imposing significant economic and societal burdens. Projected estimates indicate that by 2030 and 2050, the LTBI population will generate 16.3 and 8.3 ATB cases per 100,000 individuals, respectively [4]. Early identification of ATB patients and implementation of preventive measures to halt LTBI progression into ATB represent critical measures for controlling and eliminating TB [5]. However, the absence of specific biomarkers to identify LTBI subpopulations at risk of ATB progression remains a major challenge in TB prevention [6]. Thus, there is an urgent need to develop advanced molecular tools for TB risk stratification.

Inflammation is a fundamental response of the human immune system to signals resulting from tissue damage or pathogenic infection [7]. This process is crucial for promoting the restoration of body balance after trauma or infection by repairing damaged tissues and protecting the host from exogenous pathogens [8]. Inflammatory disorders are associated with numerous diseases, such as cancer, sepsis, and autoimmune diseases [9]. Similarly, LTBI's progression to ATB is typically accompanied by an inflammatory reaction that can reflect the development of TB [10, 11]. Following *Mycobacterium tuberculosis* infection, a “tug-of-war” between proinflammatory and anti-inflammatory signals ensues in the lung, which can promote or limit bacterial transmission [12, 13]. A shift toward a proinflammatory state can cause remodeling in granulomas, cheese liquefaction, and destruction of the surrounding lung parenchyma, all of which are related to ATB onset and the successful transmission of *Mycobacterium tuberculosis* [14, 15]. Furthermore, the regression of granuloma and pulmonary inflammation indicates a better prognosis [16]. Biomarkers related to inflammation can assist in differentiating between LTBI and ATB and predicting TB's progress. Therefore, their use can be helpful for differential diagnosis and risk stratification of TB.

Machine learning has become a powerful tool in disease research for various purposes including cancer classification and treatment, drug discovery, gene/protein interaction network analysis, and protein secondary structure prediction [17, 18]. In recent years, machine learning has been increasingly employed to identify genes with diagnostic potential, resulting in significant improvements in the accuracy of identifying differentially expressed genes on microarrays. For instance, Lee et al. [19] employed the least absolute contraction and selection operator (LASSO) algorithm to identify genes with high predictive value for treatment response after the first real flare. Similarly, Zhao and Si [20] utilized the Lasso and support vector machine recursive feature elimination (SVM-RFE) algorithms to identify key diagnostic genes for dermatomyositis. Thus, machine learning holds promise in identifying inflammatory response-related genes with significant implications in the progression of LTBI to ATB.

In this study, we employed three machine learning algorithms, namely Lasso, random forest (RF), and SVM-RFE, to identify characteristic genes associated with TB progression and inflammatory response. This allowed us to effectively distinguish between LTBI and ATB, as well as predict the progress of TB. We further investigated the role of these characteristic genes in the development of LTBI to ATB and constructed a miRNA–gene regulatory network to elucidate the underlying mechanisms. Moreover, we predict the effective drugs for characteristic genes and verify them via molecular docking.

## 2. Materials and Methods

**Figure 1:**
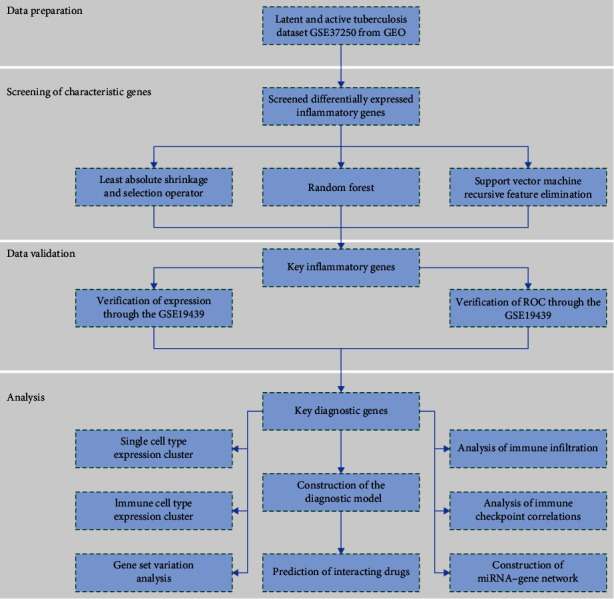
Flowchart of the design and evaluation of this study. GEO, Gene Expression Omnibus; ROC, receiver operating characteristic curve; miRNA, microRNA.

The workflow of the analysis, including the gene extraction curation pipeline, is presented in Figure 1. Our work comprises four main parts: data preparation, data processing using different machine learning models to identify characteristic genes, validation of these characteristic genes, and analysis of differences in the key characteristic genes. We elaborate on each step in the following subsections.

### 2.1. Acquisition of Microarray Data

The gene expression datasets GSE37250 [21] and GSE19439 [22] pertaining to TB were obtained from the NCBI GEO (https://www.ncbi.nlm.nih.gov/geo/). GSE37250 comprises data from 97 patients with LTBI and 83 patients with ATB, while GSE19439 comprises data from 17 LTBI patients and 13 ATB patients. GSE37250 was utilized as the training dataset, and GSE19439 was utilized as an external validation dataset.

### 2.2. Analysis of Inflammatory Response-Related Genes in the Progression of TB

Two hundred inflammatory response-related genes were obtained through the online website GSEA (https://www.gsea-msigdb.org/gsea/index.jsp) [23]. The training dataset underwent log2 transformation and normalization. Differentially expressed inflammatory response-related genes were screened with 97 LTBI and 83 ATB in accordance with the criterion that a *p*-value < 0.05 was considered significantly different, using the *limma* package [24].

### 2.3. Functional-Enrichment Analysis

Enrichment analysis was conducted on the differentially expressed inflammatory-related genes in both LTBI and ATB groups. This analysis included Gene Ontology (GO), the Kyoto Encyclopedia of Genes and Genomes (KEGG), and Disease Ontology (DO). GO enrichment analysis comprised biological processes (BP), molecular functions (MF), and cellular components (CC).

### 2.4. Selection of Characteristic Genes

The LASSO, RF, and SVM-RFE were utilized to identify characteristic genes [25]. As a dimension reduction technique, the LASSO regression demonstrates superior performance in evaluating high-dimensional data as compared with regression analysis. It employs regularization to enhance prediction accuracy [26]. R package “glmnet” were applied for LASSO, which was performed by 10-fold cross-validation to adjust the optimal penalty parameter *λ* [27]. The response type was set as binomial, and the *α* was set as 1. RF is a supervised machine learning algorithm built with a decision tree algorithm and is used to solve regression and classification problems. The “randomForest” package was used to build the RF model. The RF model was established to find the number of random forest trees with the minimum error (option trees = 148). The feature importance was determined by the mean decrease Gini index calculated by RF, and genes with relative importance >2 were determined as characteristic genes. SVM-RFE is a novel method for pattern recognition that adopts the principle of structural risk minimization, accounts for training error and generalizability, and demonstrates distinctive advantages in solving small samples, high-dimensional nonlinearity, local minima, and other pattern recognition problems [28]. R packages “e1071” and “caret” for the SVM-RFE algorithm were used to calculate the point with the smallest cross-validation error, so as to screen characteristic genes. The characteristic genes screened by the three different machine learning algorithms were then applied for feature selection by using the online tool Venny 2.1 (https://bioinfogp.cnb.csic.es/tools/venny/index.html).

### 2.5. Expression Verification and Diagnostic Effect of Characteristic Genes

Differential expression of characteristic genes in LTBI and ATB was confirmed by using the training dataset GSE37250 and the external validation dataset GSE19439. Receiver operating characteristic (ROC) curves and the area under the curve (AUC) were used for estimating the diagnostic efficacy.

### 2.6. Construction and Verification of Diagnostic Nomogram

A nomogram was constructed using the characteristic genes to predict and diagnose ATB in patients with TB. The effectiveness of the nomogram was assessed by evaluating the predictive accuracy using calibration curve analysis. Moreover, decision curve analysis and clinical impact curves were utilized to evaluate the clinical usefulness of the model.

### 2.7. Expression-Cluster Analysis

Expression clusters of characteristic genes in single cell types and immune cells were analyzed using The Human Protein Atlas online web platform (https://www.proteinatlas.org).

### 2.8. Gene-Set-Variation Analysis (GSVA)

The dataset GSE37250 was stratified into two groups based on the expression levels of characteristic genes, namely high and low, followed by pathway enrichment analysis to identify the enriched pathways associated with TB progression [29].

### 2.9. Analysis of Immunity

The ssGSEA method was utilized to calculate immune cell scores, followed by a differential analysis of immune cells and an analysis of the correlation between characteristic genes and immune cells. Furthermore, the correlation between the characteristic genes and the upregulated immune checkpoint in ATB was analyzed.

### 2.10. Construction of miRNA-Characteristic Gene Regulatory Network

Bioinformatics tools, including databases and prediction algorithms such as TargetScan (https://www.targetscan.org/vert_80/), were used to predict the miRNAs that regulate the characteristic genes [30]. The predicted miRNAs were cross-referenced using the online network tool jvenn (https://jvenn.toulouse.inra.fr/app/example.html) [31] and an interaction network between the characteristic genes and the predicted miRNAs was constructed using Cytospace.

### 2.11. Screening of Interacting Drugs

The online tool Enrichr (https://maayanlab.cloud/Enrichr/) was used to predict the five characteristic genes, and the effective drugs that played an important role in these characteristic genes were screened [32].

### 2.12. Molecular Docking

The 2D structure of retinoic acid was obtained from the PubChem database (https://pubchem.ncbi.nlm.nih.gov/). The 2D structure was converted to a 3D conformational space with minimum free energy using Chembio3D 14.0.0.117 software and the resulting file format was converted. After inputting the characteristic gene into the UniProt database (https://www.uniprot.org/), the corresponding human protein UniProt ID was selected and then used to retrieve the protein 3D structure from the RCSB PDB database (https://www.rcsb.org) [33, 34]. The protein files obtained were dehydrated, hydrogenated, and small molecular ligands were removed. Molecular docking was performed using AutoDock Vina 1.1.2 software to obtain the minimum molecular docking binding energy of the characteristic genes and the selected drugs. The hydrophobicity of the docking strength, hydrogen bond, *π*–*π* conjugated bond, and amino acid residue were analyzed using PyMOL 2.1.1 software. Finally, the best binding degree was selected and the results were presented.

### 2.13. Consensus-Clustering Analysis

K-means clustering was used on the basis of expression profiling of TB and inflammatory response-related genes (50 iterations and resampling rate of 80%) [25]. Cumulative distribution function (CDF) plots were used to find the optimal number of clusters and the relative alterations in area under the CDF curve were evaluated. Principal component analysis (PCA) was utilized to reduce dimensions and verify the reliability of consensus clusters.

## 3. Results

### 3.1. Identification of Inflammation-Related Genes Involved in the Progress of TB

**Figure 2:**
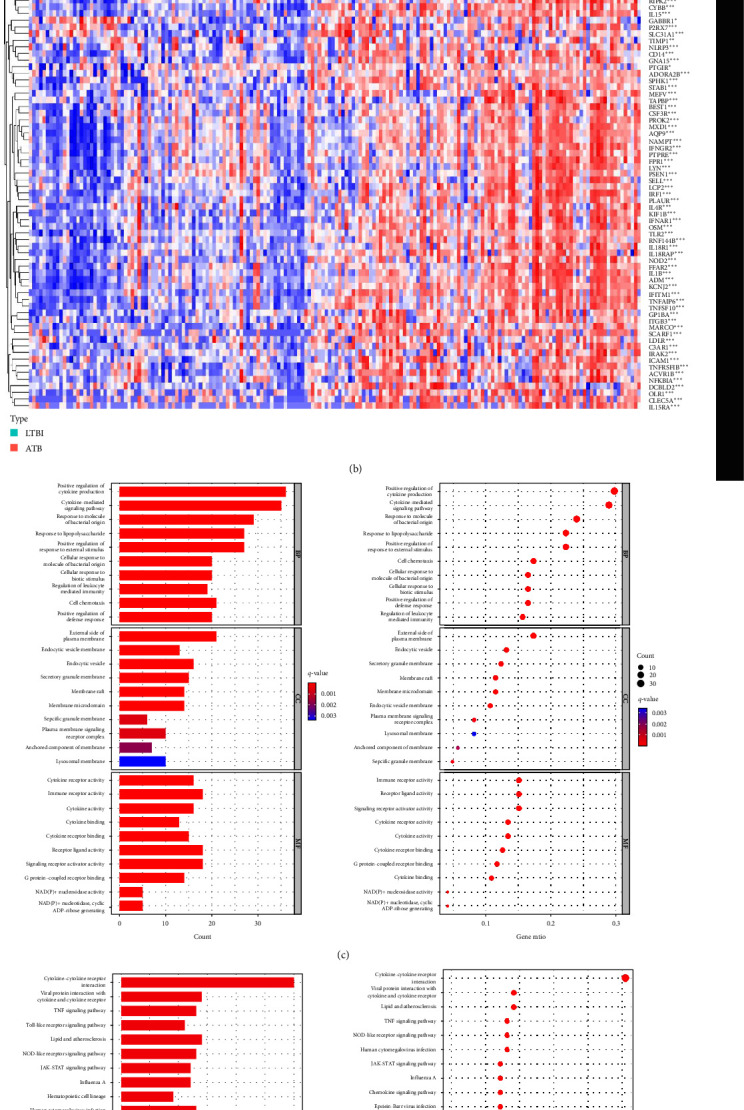
Analysis of differentially expressed inflammatory response-related genes. (a) Violin map, (b) heatmap, (c) GO analysis, (d) KEGG analysis, and (e) DO analysis of differentially expressed inflammatory response-related genes in LTBI and ATB.  ^*∗*^*P* < 0.05,  ^*∗∗*^*P* < 0.01,  ^*∗∗∗*^*P* < 0.001.

In the training dataset GSE37250, we studied the function of inflammation-related genes in the progression of TB by analyzing blood expression profiles of 97 patients with LTBI and 83 patients with ATB. Out of 200 inflammation-related genes, 122 inflammatory response-related genes showed differential expression in ATB compared with LTBI (Figure 2(a)). Among these, 96 genes were upregulated while 26 genes were downregulated (Figure 2(b)).

### 3.2. Enrichment Analysis of Differentially Expressed Inflammation-Related Genes

The enrichment analysis was implemented on these differentially expressed inflammatory response-related genes. As is shown in the results (Figure 2(c)), in terms of BP, genes are mainly enriched in the positive regulation of cytokine production, cytokine-mediated signaling pathway, and response to molecules of bacterial origin. In terms of CC, genes are mainly enriched in external side of the plasma membrane, endocytic vesicle membrane, and endocytic vesicle. In terms of MF, genes are mainly enriched in cytokine receptor activity, immune receptor activity, and cytokine activity. KEGG analysis results showed that the gene was mainly enriched in cytokine–cytokine receptor interaction, TNF signaling pathway, Toll-like receptor signaling pathway, and NOD-like receptor signaling pathway (Figure 2(d)). DO results (Figure 2(e)) showed that these genes were mostly enriched in lung diseases and bacterial infection diseases, which further indicated that these differential inflammatory response-related genes may be involved in lung diseases caused by bacteria.

### 3.3. Screening of Characteristic Genes

**Figure 3:**
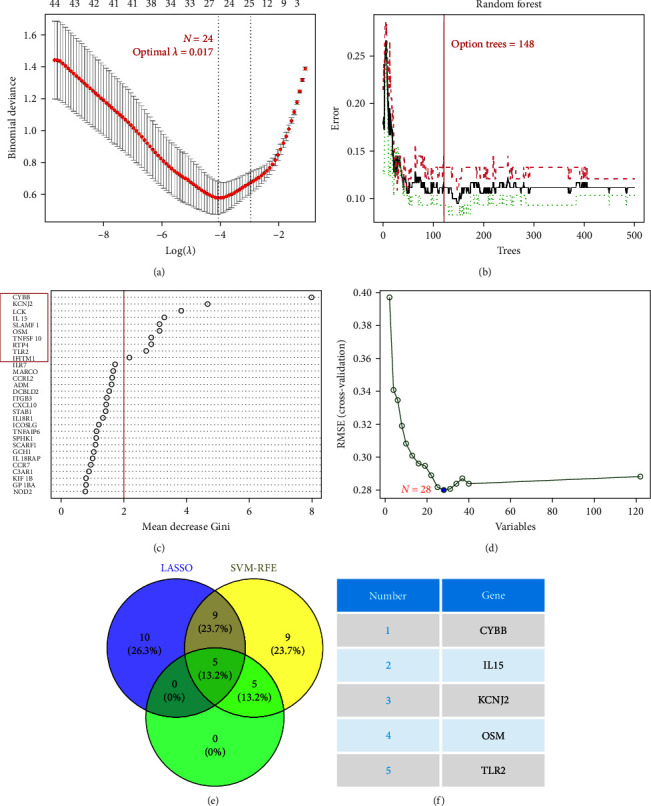
Machine learning is used to screen disease-characteristic genes. (a) Lasso algorithm for characteristic gene selection. (b) Determination of the number of decision trees in random forest algorithm. (c) Genetic importance score in random forest algorithm. (d) SVM-RFE algorithm for characteristic gene selection. (e) Venn diagram of shared characteristic genes selected by three different algorithms. (f) Shared characteristic genes in three different algorithms.

For the LASSO algorithm (Figure 3(a)), the optimal *λ* was 0.017 following 10-fold cross-validation and 24 characteristic genes were identified, containing BTG2, C3AR1, CCL2, CD14, CXCL10, CYBB, DCBLD2, EBI3, EMP3, F3, ICAM1, IL15, IL18RAP, ITGB3, KCNJ2, KLF6, OSM, SCARF1, SGMS2, SLC4A4, SPHK1, TLR2, TNFRSF1B, and TNFRSF9. For the RF algorithm (Figures 3(b) and 3(c)), the minimum error of the RF model was achieved when the number of random forest trees was 148 (Figure 3(b)).  Figure 3(c) shows the relative importance ranking of genes, among which 10 genes, including CYBB, KCNJ2, LCK, IL15, SLAMF1, OSM, TNFSF10, RTP4, TLR2, and IFITM1, were determined as characteristic genes based on their relative conditional importance score >2. The SVM-RFE algorithm (Figure 3(d)) achieved the minimum error in classification when *N* = 28. The algorithm determined 28 characteristic genes, including CYBB, KCNJ2, IL15, LCK, TLR2, TNFSF10, OSM, IL7R, RTP4, IFITM1, SLAMF1, MARCO, TNFAIP6, ITGB3, IL18RAP, SPHK1, DCBLD2, STAB1, EMP3, SCARF1, CXCL10, CCRL2, KIF1B, ICAM1, KLF6, GABBR1, FZD5, and GP1BA, as the most important features for classification. Following the intersection (Figure 3(e)), five characteristic genes shared by LASSO, random forest, and SVM-RFE algorithms were finally identified (CYBB, IL15, KCNJ2, OSM, and TLR2; Figure 3(f)). The feature genes selected by using three machine-learning methods can also be found in Table S1.

### 3.4. Expression Verification and Diagnostic Effect of Characteristic Genes

**Figure 4:**
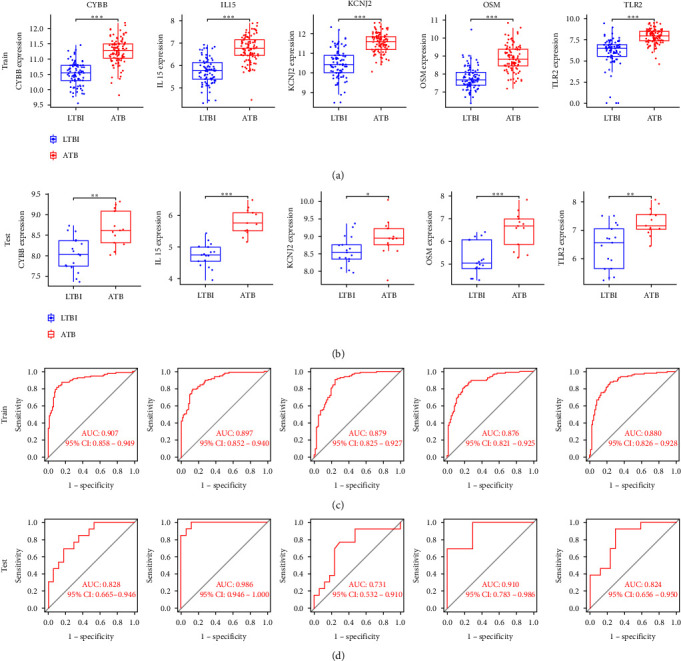
Expression verification and diagnostic effect of characteristic genes. The expression of characteristic genes in (a) training dataset GSE37250 and (b) external test dataset GSE19439. The diagnostic performance for ATB of characteristic genes in (c) training data set GSE37250 and (d) external test dataset GSE19439.  ^*∗*^*P* < 0.05,  ^*∗∗*^*P* < 0.01,  ^*∗∗∗*^*P* < 0.001.

In the training dataset GSE37250 and the external test dataset GSE19439, five characteristic genes (CYBB, IL15, KCNJ2, OSM, and TLR2) presented higher expression in ATB than that in LTBI (Figures 4(a) and 4(b)), indicating their potential role in the development of TB. We also estimated the diagnostic performance of each characteristic gene for ATB in GSE37250 and GSE19439, respectively (Figures 4(c) and 4(d)). The AUC values of ROC curves were greater than 0.7, demonstrating that these characteristic genes enabled to distinguish ATB from LTBI. Therefore, the characteristic genes have excellent diagnostic performance in predicting the progress of TB.

### 3.5. Establishment of Nomogram Based on Characteristic Genes to Predict the Progress of TB

**Figure 5:**
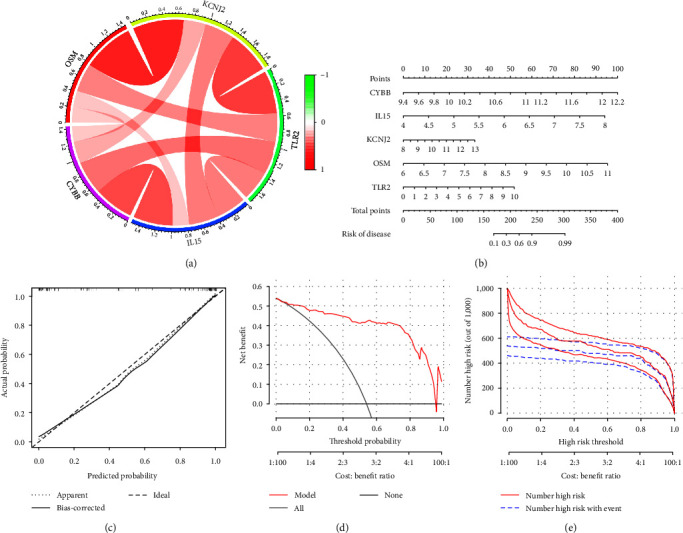
Diagnostic nomogram based on characteristic gene and its verification. (a) Interactions between characteristic genes at the molecular level. (b) Establishment of a nomogram integrating characteristic genes for predicting TB progression. (c) The calibration curve estimates the prediction accuracy of the nomogram. (d) Decision curve analysis shows the clinical benefit of the nomogram. (e) The clinical influence curve shows the clinical practicability of the nomogram.

As illustrated in Figure 5(a), there were significant interactions between the characteristic genes. By incorporating these characteristic genes, a nomogram was constructed as a diagnostic tool for ATB, and its predictive abilities were evaluated via calibration curves. In the nomogram (Figure 5(b)), each characteristic gene corresponded to a score, and the total score was calculated by adding the scores for all the characteristic genes. The total points corresponded to different risks of TB. The calibration curve demonstrated that the difference between the actual and predicted risks of ATB was very small, indicating that the nomogram enabled an accurate estimation of the progression of TB (Figure 5(c)). As depicted in the decision curve analysis (Figure 5(d)), the nomogram model was found to be highly accurate. The clinical influence curve (Figure 5(e)) showed that the predicted ATB by the nomogram was consistent with the actual situation.

### 3.6. Expression-Cluster Analysis of Characteristic Genes

**Figure 6:**
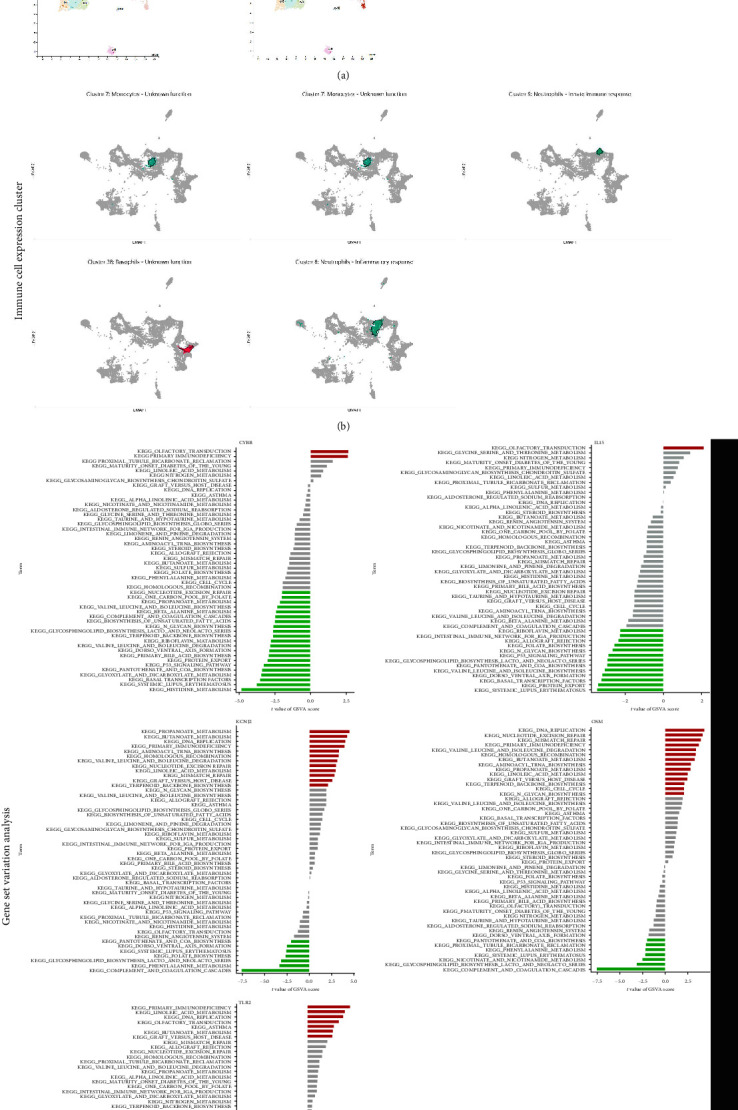
Expression cluster and GSVA analysis of characteristic genes. (a) single cell expression cluster analysis, (b) immune cell expression cluster analysis, and (c) GSVA analysis of characteristic genes.

The expression of characteristic genes was evaluated via expression cluster analysis. As shown in the single-cell expression cluster analysis (Figure 6(a)), CYBB, IL15, KCNJ2, and TLR2 were predominantly expressed in macrophages, while OSM was primarily expressed in T cells. CYBB and IL15 were mainly expressed in monocytes, KCNJ2 was mainly expressed in neutrophils, and OSM was mainly expressed in basophils (Figure 6(b)). The identification of these cell types and their gene expression patterns is of significant value in understanding the roles of macrophages, T cells, neutrophils, and basophils during the progression of TB.

### 3.7. Gene-Set-Variation Analysis

To gain a better understanding of the role of characteristic genes in TB, we conducted a GSVA to classify TB into two subgroups based on the median expression of characteristic genes (Figure 6(c)). Primary immunodeficiency and olfactory transduction pathways were significantly enriched in the high CYBB subgroup, while histidine metabolism, systemic lupus erythematosus, and basal transcription factor were significantly enriched in the low CYBB subgroup. Olfactory transduction was significantly enriched in the high IL15 subgroup, while systemic lupus erythematosus, protein output, and basal transcription factor were significantly enriched in the low IL15 subgroup. Propanoate metabolism, butanoate metabolism, DNA replication, and primary immunodeficiency were significantly enriched in the high KCNJ2 subgroup, while the complement and cohesive cascades, phenylalanine metabolism, and biosynthesis of lactose and neolactone series of glycosphingolipids were significantly enriched in the low KCNJ2 subgroup. The high OSM subgroup was highly enriched in DNA replication, nucleotidectomy repair, and primary immunodeficiency, while the low OSM subgroup was significantly enriched in supplement and solidification cascade, biosynthesis of lactose and neolactone series of glycosphingolipids, and metabolism of nicotinate and nicotinamide. Primary immunodeficiency, linoleic acid metabolism, and DNA replication were significantly enriched in the high TLR2 subgroup, while pantothenate and CoA biosynthesis, biosynthesis of lactose and neolactone series of glycosphingolipids, and the complement and cohesive cascade were significantly enriched in the low TLR2 subgroup. Among these enriched pathways, primary immunodeficiency was associated with the highest expression of characteristic genes, indicating a prominent role in the progression of TB.

### 3.8. Changes in Immunological Characteristics from the LTBI and ATB using ssGSEA Analysis

**Figure 7:**
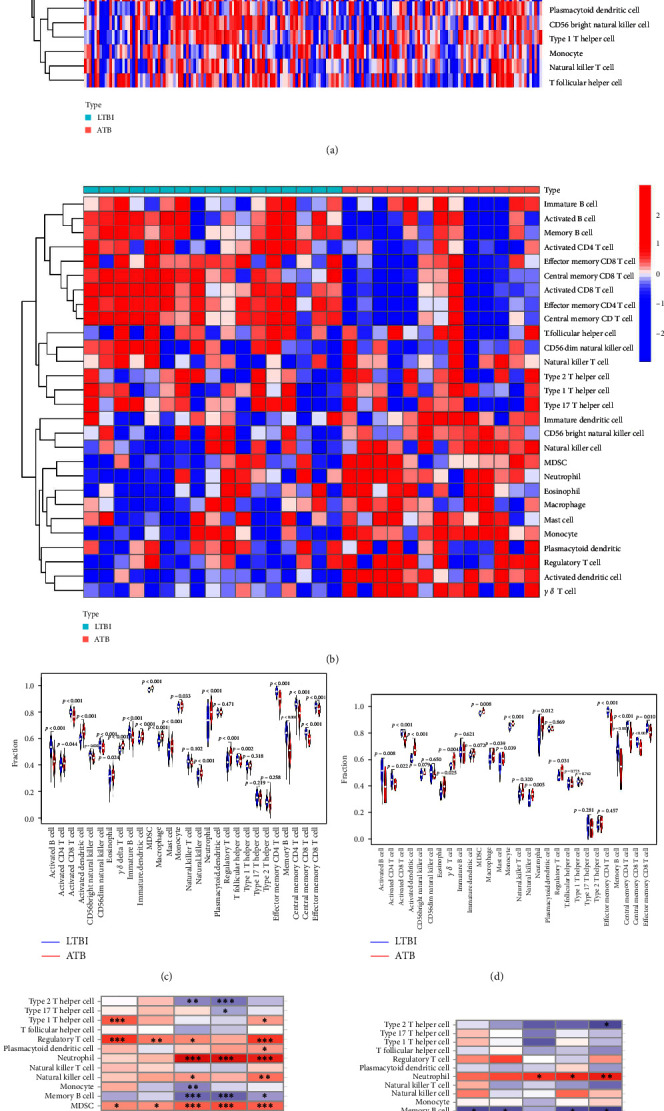
Analysis of immune infiltration. The heatmaps of immune cells in (a) training dataset GSE37250 and (b) external test dataset GSE19439. The violin diagram of immune cells in (c) training dataset GSE37250 and (d) external test dataset GSE19439. The correlation between characteristic genes and immune cells in (e) training dataset GSE37250 and (f) external test dataset GSE19439.

ssGSEA was used to analyze the changes in immunological characteristics from LTBI to ATB and investigate the relationship between immune infiltration and ATB/LTBI. The analysis was conducted using a training dataset and an external test dataset. Figures 7(a) and 7(b) depict immune cell heatmaps in the training dataset GSE37250 and the test dataset GSE19439, respectively. Figures 7(c) and 7(d) showed the violin diagram of immune cell expression in the training dataset GSE37250 and the test dataset GSE19439 respectively. The results (Table S2) demonstrate that in the training dataset, 23 immune cells exhibited differential expression, with 12 immune cells upregulated and 11 immune cells downregulated. In the external test dataset, 18 immune cells showed differential expression, with 10 immune cells upregulated and 8 immune cells downregulated. The trend of immune cell differential expression was consistent in both the GSE37250 and GSE19439 datasets, with 10 immune cells upregulated. Furthermore, we analyzed the relationship between characteristic genes and immune cells, as shown in Figures 7(e) and 7(f) for the training and test validation datasets, respectively. It was observed that upregulated immune cells and characteristic genes were mostly positively correlated, whereas downregulated immune cells and characteristic genes were mostly negatively correlated. These findings suggest that the characteristic genes may play a role in regulating the immune process during the development of LTBI into ATB.

### 3.9. Immune-Checkpoint Analysis

**Figure 8:**
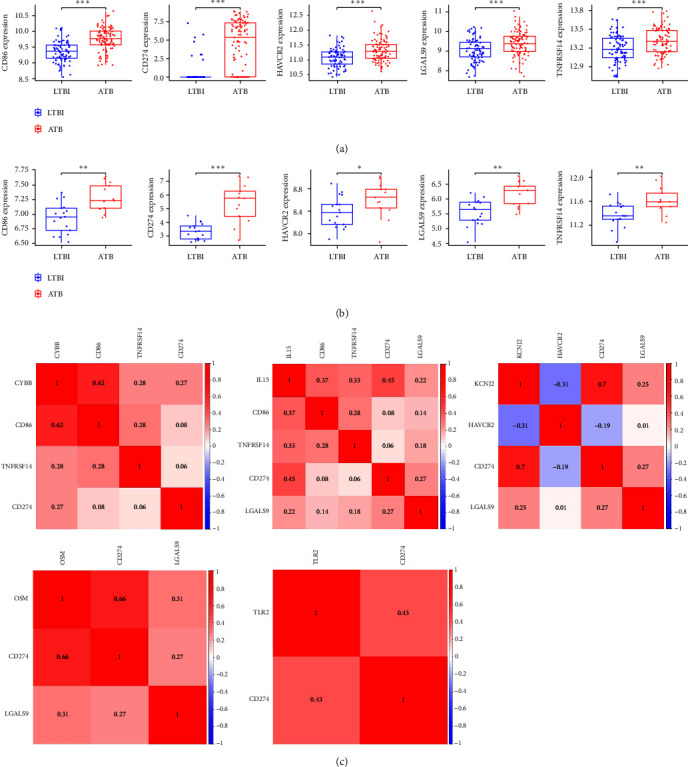
Evaluation of immune checkpoint. The expression of upregulated immune checkpoint gene in (a) training dataset GSE37250 and (b) external test dataset GSE19439. (c) The correlation between characteristic genes and immune checkpoint genes.  ^*∗*^*P* < 0.05,  ^*∗∗*^*P* < 0.01,  ^*∗∗∗*^*P* < 0.001.

To further investigate the immunological characteristics of ATB and LTBI, we analyzed changes in immune checkpoints. A total of five immune checkpoints were upregulated in both the training dataset GSE37250 (Figure 8(a)) and the external test dataset GSE19439 (Figure 8(b)). We analyzed the coexpression relationship between the five characteristic genes and these upregulated immune checkpoints (Figure 8(c)). The CYBB gene was positively linked to CD86, TNFRSF14, and CD274. The IL15 gene was positively linked to CD86, TNFRSF14, CD274, and LGALS9. The KCNJ2 gene was positively linked to CD274 and LGALS9 and negatively linked to HAVCR2. The OSM gene was positively linked to CD274 and LGALS9. The TLR2 gene was positively linked to CD274. The results indicate that the characteristic genes were mostly positively linked to the upregulated immune checkpoints in the progression of TB, and CD274 was positively linked to all five characteristic genes. Therefore, characteristic genes may be involved in the regulation of immune checkpoints in the process of TB progression, and CD274 immune checkpoint may be an effective target for inhibiting the progression of LTBI into ATB and treating ATB.

### 3.10. Construction of miRNA-Characteristic Gene Regulatory Network

**Figure 9:**
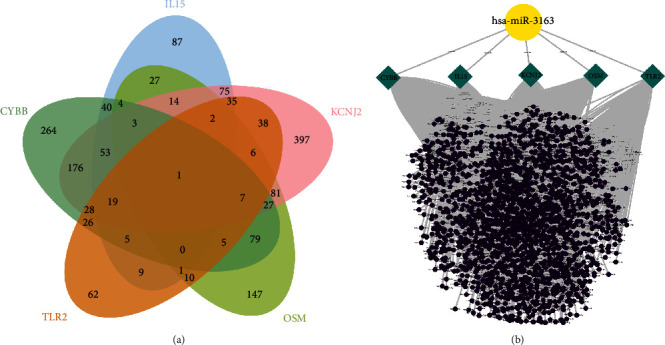
Construction of miRNAs-characteristic gene regulatory network. (a) The Venn diagram shows the miRNA shared upstream of the five characteristic genes. (b) The miRNA–genes regulatory network based on characteristic genes.

To further study the regulatory network of characteristic genes, we predicted the miRNA upstream of characteristic genes (Figure 9(a)) and constructed the network via these characteristic genes and miRNA (Figure 9(b)). The results showed that 737 miRNAs were predicted upstream of CYBB gene, 375 miRNAs were predicted upstream of IL15 gene, 962 miRNAs were predicted upstream of KCNJ2 gene, 414 miRNAs were predicted upstream of OSM gene, and 254 miRNAs were predicted upstream of TLR2 gene. In these miRNAs, hsa-miR-3163 was predicted by all characteristic genes, indicating that hsa-miR-3163 has a regulatory effect on all characteristic genes, which may be one of the important regulatory nodes in the development of LTBI into ATB.

### 3.11. Analysis of Effective Drugs

**Figure 10:**
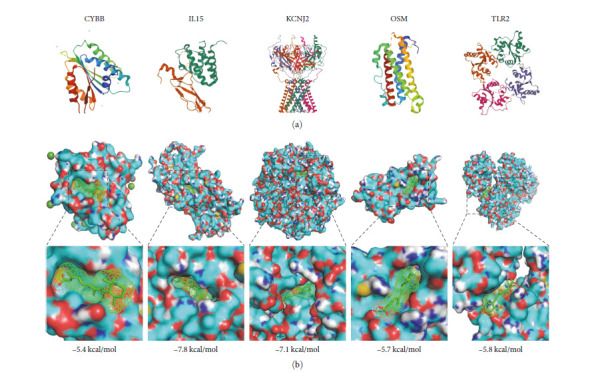
Molecular docking between small molecule drugs and corresponding proteins of characteristic genes. (a) The protein corresponding to the characteristic gene. (b) Demonstration diagram of the minimum binding energy between the small molecule drug and the corresponding protein of the characteristic gene.

On the basis of characteristic genes, we predicted the potentially effective drugs that can prevent LTBI from developing into ATB. The results showed that a total of 253 drugs (Figure S1) were predicted, of which retinoic acid could interact with the most characteristic genes. Retinoic acid was then linked to the proteins of the five characteristic genes. The five characteristic genes were converted into the corresponding protein (Figure 10(a)), and then the binding form (Figure 10(b)) with the lowest binding energy was obtained by docking retinoic acid with the corresponding protein. The binding site of retinoic acid and the protein was amplified. The results showed that retinoic acid enabled to dock with five proteins, and the gene binding energy of CYBB, IL15, KCNJ2, OSM, and TLR2 genes was −5.4, −7.8, −5.7, and −5.8 kcal/mol, respectively. The above results effectively demonstrated the reliability of predicting drugs and showed that small-molecule retinoic acid may play a potential role in preventing LTBI from developing into ATB.

### 3.12. Construction of Two Inflammatory Subtypes of TB Based on Inflammatory Response-Related Genes

**Figure 11:**
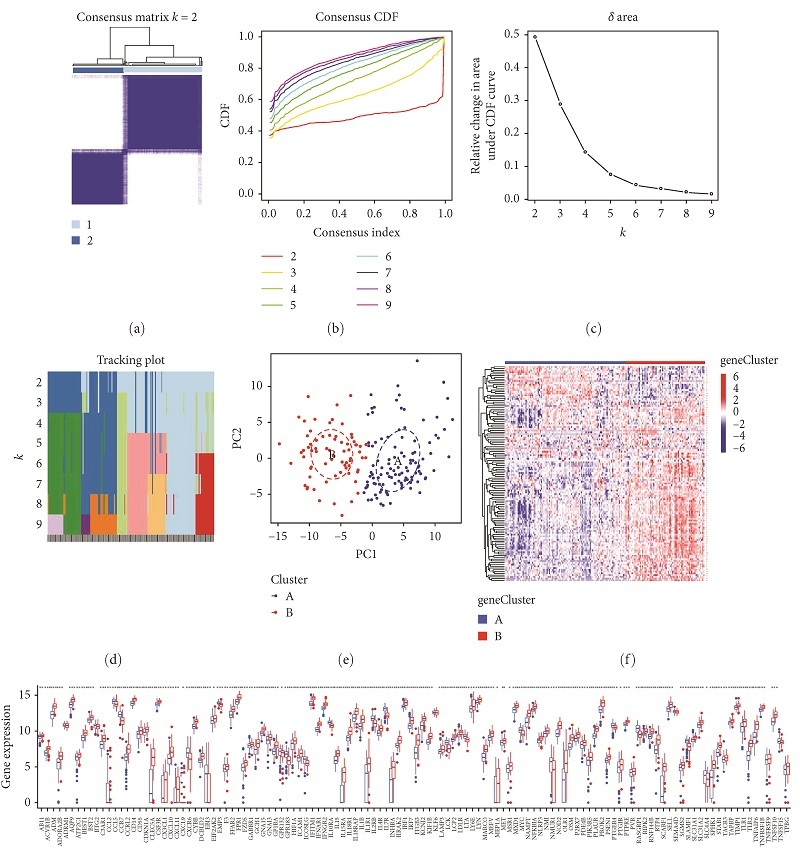
Construction of two inflammation subtypes of TB based on the TB progress and inflammatory response-related genes. (a) Consensus matrix heatmap when *k* = 2. (b) Consensus CDF when *k* = 2–9. (c) Relative alterations in the area under the CDF curve. (d) Tracking plot showing the sample classification when *k* = 2–9. (e) PCA plots demonstrating that TB specimens are categorized as two inflammation subtypes (A, B). (f) Heatmap showing the expression of TB progression- and inflammatory response-related genes in two inflammation subtypes. (g) Violin diagram of TB progression and inflammatory response-related genes.  ^*∗*^*P* < 0.05,  ^*∗∗*^*P* < 0.01,  ^*∗∗∗*^*P* < 0.001.

TB was clustered via the consensus clustering method based on the progression of TB and the expression profile of genes related to inflammatory response. The optimal number of subtypes was found to be 2, as determined by a consensus matrix plot (Figure 11(a)), a CDF plot (Figure 11(b)), relative alterations in the area under the CDF curve (Figure 11(c)), and a tracking plot (Figure 11(d)). We named the two immune subtypes A and B. PCA analysis demonstrated a remarkable difference between the subtypes (Figure 11(e)). Notably, significant heterogeneity existed in subtypes of TB and the expression of genes related to inflammatory response (Figure 11(f)). Furthermore, we observed that all characteristic genes presented higher expression in subtype B than A, and most genes related to inflammatory response exhibited higher expression in subtype B than A (Figure 11(g)). Therefore, we identified subtype B as an immune subtype and subtype A as a nonimmune subtype.

## 4. Discussion

According to experimental and clinical evidence, TB is a significant infectious disease globally, leading to severe social and economic burdens [35–37]. Early identification of LTBI and ATB has become the key to control and eliminate TB. Early detection of LTBI and ATB is crucial for effective control and elimination of TB. Unfortunately, no highly effective biomarker is available to identify LTBI and ATB or to predict the progression of TB. Therefore, it is crucial to develop innovative diagnostic tools for risk stratification of TB.

In the expression profile of individuals with LTBI and ATB from the GSE37250 dataset, we have identified 122 inflammatory response-related genes that exhibit differential expression in individuals with ATB compared with those with LTBI. Enrichment analysis was performed on these genes, which showed that they were primarily involved in the positive regulation of cytokine production, the external side of the plasma membrane, and cytokine receptor activity, as revealed by GO analysis. KEGG pathway analysis indicated that these inflammatory response-related genes were primarily associated with cytokine–cytokine receptor interaction, TNF signaling pathway, Toll-like receptor signaling pathway, and NOD-like receptor signaling pathway. DO analysis indicated that these inflammatory response-related genes were mainly enriched in lung diseases and bacterial infections, further highlighting their involvement in TB-induced lung diseases. These findings underscore the crucial role of inflammatory response-related genes in the development and progression of TB and may pave the way for the development of novel diagnostic tools for TB.

Three algorithms were employed to identify characteristic genes from inflammatory response-related genes involved in the progression of TB. The research findings demonstrate that these characteristic genes play a crucial role in TB progression. The CYBB gene encodes the gp91-phox component of the phagocytic oxidase complex, which is responsible for producing superoxide and other downstream reactive oxygen species (ROS) critical for microbial killing [38, 39]. For a long time, ROS has been considered to be the primary cause of tissue damage resulting from acute or chronic inflammatory diseases in pathological conditions [40, 41]. Therefore, the upregulation of CYBB indicates an excess of ROS, which may contribute to tissue injury and clinical symptoms arising from LTBI progression ATB. IL-15 is an inflammatory cytokine that plays a significant role in the development and functional maturation of T lymphocytes and natural killer cells [42–44]. IL-15 has been shown to be involved in various autoimmune inflammatory diseases [45–47]. It can promote chronic inflammation and sustain the inflammatory process. Moreover, viral infections can upregulate IL-15 expression [48]. The protein encoded by the KCNJ2 gene is a complete membrane protein and inward rectifier potassium channel that conducts strong inward rectifier K current in various tissues and cell types, including neurons, skeletal muscle, cardiomyocytes, immune system, and cancer cells [49]. Voltage-dependent potassium channels are one of the critical regulatory factors in the maturation, activation, and differentiation of macrophages, playing a key role in macrophage proliferation and activation by affecting the resting potential balance of macrophages [50, 51]. Therefore, the upregulation of KCNJ2 may indicate an increase in macrophages. OSM is a member of the interleukin-6 cytokine family. Inflammatory cells can infiltrate specific microenvironments and secrete OSM, which binds to the extracellular matrix and helps to create an inflammatory microenvironment [52, 53]. TLR are recognition molecules for a variety of pathogens, including bacteria, viruses, fungi, and parasites [54]. In TB, TLR2 contributes to the pathological scope and spatial localization of infected lung tissue [55].

In order to elucidate the role of characteristic genes in TB progression, we conducted a series of analyses of characteristic genes. Our findings indicate that these genes are primarily expressed in macrophages, T cells, neutrophils, and basophils. The subgroup of characteristic genes associated with primary immunodeficiency showed the highest levels of expression and played a prominent role in the disease progression. Additionally, we confirmed the key role of these characteristic genes in TB through expression verification and diagnostic validity analyses of external datasets. Our immune analysis further revealed that most of the upregulated immune cells in the transition from LTBI to ATB were positively correlated with the characteristic genes, while the downregulated immune cells showed negative correlation. This suggests that the characteristic genes may regulate the immune process during the development of LTBI into ATB. We also observed a positive correlation between most of the characteristic genes and the upregulated immune checkpoint during TB progression. Furthermore, CD274 showed a positive correlation with five characteristic genes simultaneously, implying that the characteristic genes may be involved in the regulation of immune checkpoints during TB progression. Our results suggest that CD274 immune checkpoint may be an effective target to inhibit the progression of LTBI into ATB and to treat ATB.

The upstream miRNA and effective drugs were predicted by characteristic genes to find out the key action points and drugs that can effectively prevent the progress of LTBI from developing into ATB. Hsa-miR-3163 is a miRNA that can regulate all five characteristic genes, which may be an important network node in the molecular mechanism of LTBI developing into ATB. Retinoic acid interacts with the most characteristic genes. The results of molecular docking showed that the binding energy of retinoic acid to the proteins corresponding to the characteristic genes was less than −5.0 kcal/mol, which indicated that retinoic acid might prevent LTBI from developing into ATB, and was helpful in the treatment of ATB. Finally, we constructed two subtypes on the basis of the expression profile of inflammatory response-related genes related to TB progress. Further analysis demonstrated that the B immune subtypes showed higher expression of inflammatory response-related genes than the A noninflammatory subtypes. Therefore, our classification enables us to reflect the inflammatory landscape of TB, which may contribute to the early diagnosis and intervention of TB treatment.

## 5. Conclusion

Collectively, our work has found the key characteristic genes in the development of LTBI into ATB, and these characteristic genes have been used to establish diagnostic lines. Subsequently, a series of analyses of these characteristic genes were carried out, which may help us to broaden our understanding of the molecular mechanism and bring more potential therapeutic targets for the clinic. Retinoic acid may play a role in preventing LTBI from progressing to ATB and treating ATB. Our study may provide a potential basis and feature direction for the differential diagnosis of LTBI and ATB and the progress mechanism and prevention of TB.

## Data Availability

The datasets presented in this study can be found in online repositories. The names of the repository/repositories and accession number(s) can be found in the article.

## Supplementary Materials

Supplementary Materials Figure S1: A network map of characteristic genes and effective drugs. Table S1: The characteristic genes were selected using three different machine learning methods. Table S2: The expression trends of immune cells in datasets GSE37250 and GSE19439.
