# Supplementary material for: Predicting the Progress of Tuberculosis by Inflammatory Response-Related Genes Based on Multiple Machine Learning Comprehensive Analysis
Source: J Immunol Res. 2023 May 16;2023:7829286. doi: 10.1155/2023/7829286 (PMC10205410; doi:10.1155/2023/7829286)
Supplement: Supplementary Materials — Figure S1: A network map of characteristic genes and effective drugs. Table S1: The characteristic genes were selected using three different machine learning methods. Table S2: The expression trends of immune cells in datasets GSE37250 and GSE19439. [file 7829286.f1.docx]

**Predicting the progress of tuberculosis by inflammatory response-related genes based on multiple machine learning comprehensive analysis.**

**Shuai Ma^1†^, Peifei Peng^2^, Zhihao Duan^1^, Yifeng Fan^1^, Xinzhi Li^1^***

^1^Hubei Key Laboratory of Tumor Microenvironment and Immunotherapy, China Three Gorges University; College of Basic Medical Science, China Three Gorges University, Yichang 443000, China;

^2^Department of Geriatrics, Liyuan Hospital, Tongji Medical College, Huazhong University of Science and Technology, Wuhan, Hubei 430074;

^*^***Corresponding author:***

*Dr.* Xinzhi Li, Hubei Key Laboratory of Tumor Microenvironment and Immunotherapy, China Three Gorges University; College of Basic Medical Science, China Three Gorges University, Yichang 443000, China;

E-mail: [lixpj@163.com](mailto:lixpj@163.com)


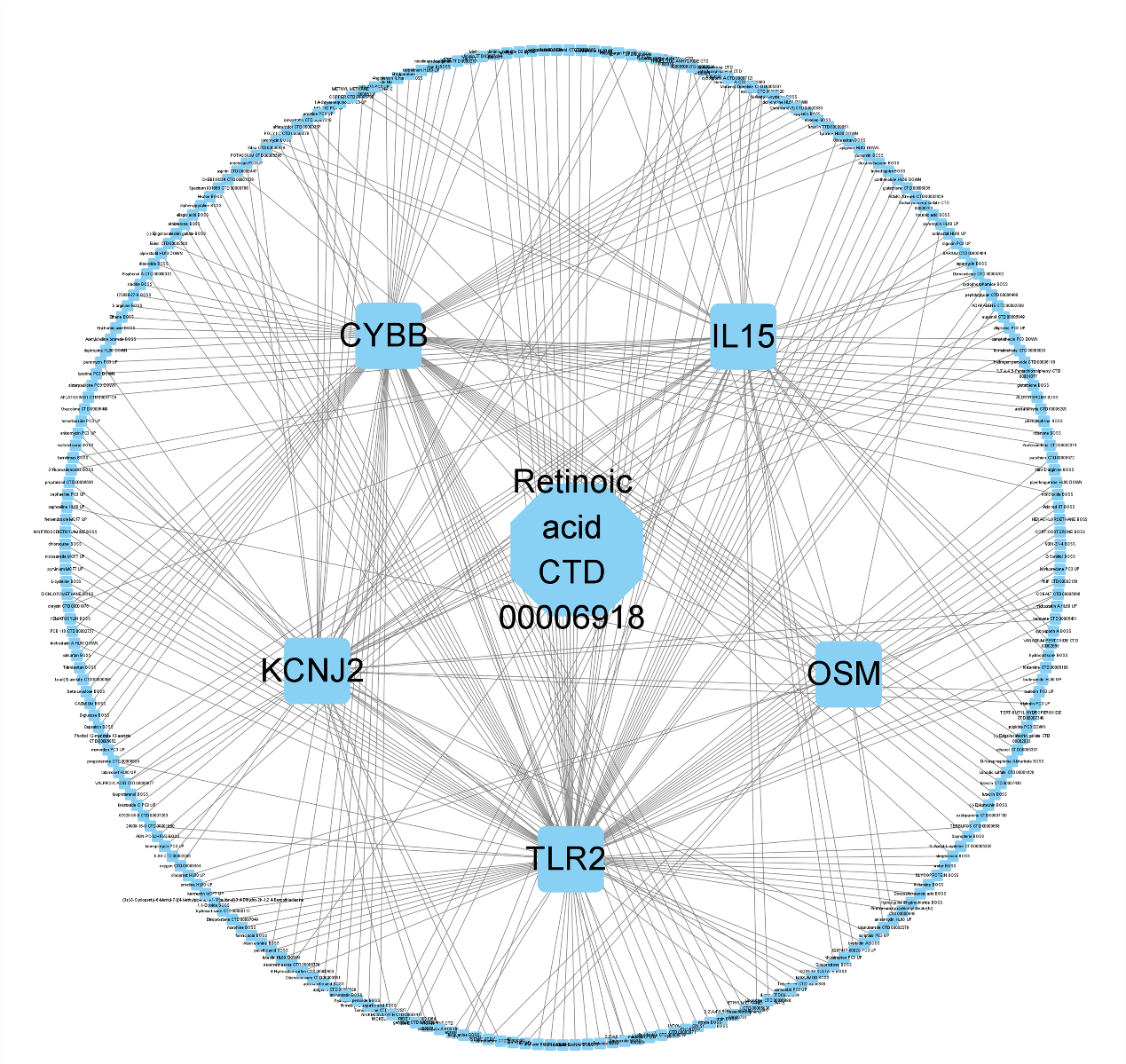


**Fig. S1** A network map of characteristic genes and effective drugs.

| Lasso | RF | SVM-RFE |
| --- | --- | --- |
| CYBB | CYBB | CYBB |
| IL15 | IL15 | IL15 |
| KCNJ2 | KCNJ2 | KCNJ2 |
| OSM | OSM | OSM |
| TLR2 | TLR2 | TLR2 |
| BTG2 | LCK | LCK |
| C3AR1 | SLAMF1 | TNFSF10 |
| CCL2 | TNFSF10 | IL7R |
| CD14 | RTP4 | RTP4 |
| CXCL10 | IFITM1 | IFITM1 |
| DCBLD2 |  | SLAMF1 |
| EBI3 |  | MARCO |
| EMP3 |  | TNFAIP6 |
| F3 |  | ITGB3 |
| ICAM1 |  | IL18RAP |
| IL18RAP |  | SPHK1 |
| ITGB3 |  | DCBLD2 |
| KLF6 |  | STAB1 |
| SCARF1 |  | EMP3 |
| SGMS2 |  | SCARF1 |
| SLC4A4 |  | CXCL10 |
| SPHK1 |  | CCRL2 |
| TNFRSF1B |  | KIF1B |
| TNFRSF9 |  | ICAM1 |
|  |  | KLF6 |
|  |  | GABBR1 |
|  |  | FZD5 |
|  |  | GP1BA |

**Supplementary Tables S1**

**Supplementary Tables S2**
